# Investigating the genetic relationship of intracranial and subcortical brain volumes with depression and other psychiatric disorders

**DOI:** 10.1162/imag_a_00291

**Published:** 2024-09-19

**Authors:** Luis M. García-Marín, Natalia S. Ogonowski, Laura K.M. Han, Mateo Maya-Martínez, Brittany L. Mitchell, Lianne Schmaal, Nicholas G. Martin, Miguel E. Rentería

**Affiliations:** Brain & Mental Health Program, QIMR Berghofer Medical Research Institute, Brisbane, QLD, Australia; School of Biomedical Sciences, Faculty of Medicine, University of Queensland, Brisbane, QLD, Australia; Centre for Youth Mental Health, The University of Melbourne, Melbourne, VIC, Australia; Orygen, Parkville, VIC, Australia; Licenciatura en Ciencias Genómicas, Centro de Ciencias Genómicas, Universidad Nacional Autónoma de México, Cuernavaca, Morelos, Mexico

**Keywords:** depression, genetics, neuroimaging, MRI, pleiotropy: GWAS

## Abstract

Depression is one of the most common mental health disorders worldwide, yet its neurobiological mechanisms remain poorly understood. Structural brain differences in subcortical limbic regions are thought to be implicated in the pathology of depression. We leveraged genome-wide association studies (GWAS) summary-level data to explore the molecular pathways underlying the relationship between genetic risk for depression and intracranial and subcortical brain volumes measured via magnetic resonance imaging. At the whole-genome level, we identified a negative genetic correlation (rG) between depression and the volume of the ventral diencephalon (rG = -0.08), which remained significant after adjusting for multiple testing. We observed nominal (*P*< 0.05) positive genetic correlations between depression and the volumes of the caudate nucleus (rG = 0.06) and the putamen (rG = 0.06), while hippocampal volume displayed a negative genetic correlation (rG = -0.06) with depression. Pairwise GWAS analyses uncovered 104 genome segments with genetic variants influencing the aetiology of depression and at least one brain volume at the local genetic level. Gene association analyses of these genomic segments suggest putative links with dopamine neurotransmission, mesocorticolimbic functional connectivity, GABAergic transmission, and the insulin signalling pathway. Sensitivity analyses showed that the volume of the ventral diencephalon is also negatively correlated with bipolar disorder and schizophrenia; however, most of the genes associated with depression and brain volumes are specific for depression and do not replicate when investigating bipolar disorder or schizophrenia with brain volumes. We observed negative phenotypic correlations between depression and intracranial and subcortical brain volumes. Overall, our findings contribute to our understanding of the neurobiology of depression and suggest that, besides the known role of the hippocampus, other subcortical structures might also play essential roles in the aetiology of depression.

## 1 Introduction

Depression is among the most common mental health disorders, with a 12-month prevalence of ~5% worldwide (Bromet et al., 2011;Gutiérrez-Rojas et al., 2020;Lim et al., 2018;World Health Organization, 2017). It is characterised by losing pleasure or interest in otherwise enjoyable activities, fatigue, sleep disturbances, and feelings of worthlessness (Aman et al., 2022;Christensen et al., 2020). The pathophysiology of depression has been a focal point of interest in neuropsychiatric research for several decades. However, the substantial heterogeneity of the disease poses significant challenges to advancing our understanding of its aetiology (Athira et al., 2020;Hasler, 2010), ultimately hindering the efficacy of potential treatment strategies (García-Marín et al., 2022;Taliaz et al., 2021).

Several neurobiological theories have been proposed to describe the pathophysiology of depression. Most of them point toward the role of psychological stress, stress hormones, neurotransmission, neurocircuitry, and circadian rhythms in the development and severity of depression (Hasler, 2010). In particular, several previous studies have investigated the associations between the human cortex and depression (Hare & Duman, 2020;Pizzagalli & Roberts, 2022;Wu et al., 2023), while some observational studies and animal models have reported smaller hippocampal volume among individuals with depression (Campbell & MacQueen, 2004;Malykhin et al., 2010;Schmaal et al., 2016;Sheline, 2011). Notably, magnetic resonance imaging (MRI) case-control studies have highlighted that the volume of subcortical brain structures tends to be lower among individuals with depression and no comorbid anxiety (Espinoza Oyarce et al., 2020). Furthermore, other studies point to the putative role of the amygdala in depression (Grogans et al., 2022;Pandya et al., 2012;Schmaal et al., 2016), which may be specific for individuals with comorbid anxiety (Espinoza Oyarce et al., 2020). Nonetheless, the relationship between depression and the intracranial and subcortical structures in the human brain remains poorly understood.

Genetic epidemiological studies indicate that depression is a heritable trait, with heritability estimates in the range of 9% for SNP-based heritability (Ormel et al., 2019) and 38% for broad sense heritability (Flint & Kendler, 2014). Similar observations have been made in neuroimaging genetic studies, which identify the genetic variants associated with the size of different brain structures measured from MRI scans. For instance, the heritability of intracranial and subcortical brain volumes ranges from 9 to 47% in genetic association studies (Nawaz et al., 2022;Satizabal et al., 2019), while twin studies report heritability estimates ranging from 25% for the volume of the amygdala to 89% for the volume of the putamen (Rentería et al., 2014). Although previous studies combining genetic and imaging data have been limited by the relatively small sample sizes available for intracranial and subcortical brain volumes (Schmaal et al., 2016;Wigmore et al., 2017), some have suggested positive genetic overlap of nominal significance (*P*< 0.05) between hippocampal volume and depression at the whole-genome level, which would suggest that genetic variants increasing the likelihood of developing a larger hippocampal volume could be associated with a higher genetic susceptibility for depression (Wigmore et al., 2017). However, only after recurrent depressive episodes or an early onset individuals would experience a reduction in hippocampal volume (Schmaal et al., 2016;Videbech & Ravnkilde, 2015;Wigmore et al., 2017).

Overall, findings from previous studies provide inconclusive associations regarding the relationship of depression with intracranial and subcortical brain volumes (Mathias et al., 2016;Videbech & Ravnkilde, 2015;Wigmore et al., 2017), perhaps due to differences in sample sizes, statistical power, and ancestral background of participants. Thus, there is a growing need to pinpoint the shared molecular pathways underlying these potential associations. In the present study, we leverage GWAS summary data for depression, intracranial, and subcortical brain volumes to investigate the genetic associations between depression and 10 brain volumes to identify molecular pathways and shared biological mechanisms influencing the aetiology of both depression and brain structural brain morphometry.

## 2 Methods

### 2.1 Ethics statement

This study used GWAS summary statistics data from previously published studies. The original studies were conducted under appropriate ethics oversight, and the details are described in the relevant publications.

### 2.2 Depression GWAS data

We leveraged GWAS summary statistics for a lifetime history of depression from the Psychiatric Genomics Consortium (PGC), UK Biobank, and 23andMe, Inc. (Howard et al., 2019). Briefly, the full set of GWAS summary statistics was obtained through an inverse-variance weighted meta-analysis conducted on participants of European ancestry from the UK Biobank (Elliott et al., 2018), the PGC (Sullivan et al., 2018), and 23andMe. The sample included 246,363 cases and 561,190 controls of European ancestry from at least 30 samples or cohorts worldwide, resulting in a total sample size of 807,553 individuals. Among the cases, ~37% were males and ~63% were females (Howard et al., 2019), while for the controls ~50% were males and ~50% were females (Howard et al., 2019). Individual genotype data were processed using the PGC ricopili pipeline or a similar procedure across cohorts (Wray et al., 2018). These GWAS summary statistics excluded variants with an imputation accuracy threshold <0.6 and with a minor allele frequency <0.005, including a total of 8,098,588 genetic variants (Howard et al., 2019).

A broad lifetime history definition of depression was used for the UK Biobank cohort (Howard et al., 2018). For instance, participants with depression were identified by the response to the question “Have you ever seen a general practitioner for nerves, anxiety, tension or depression?” or “Have you ever seen a psychiatrist for nerves, anxiety, tension or depression?” (Howard et al., 2019). In the 23andMe cohort, depression was determined via an online survey in which individuals self-reported having received a clinical diagnosis or treatment for depression (Howard et al., 2019;Hyde et al., 2016). In the PGC cohort, depression was determined by identifying individuals who met the international (DSM-IV, ICD-9, or ICD-10) for a lifetime diagnosis of major depressive disorders (Wray et al., 2018). The assessment was performed by professionally trained interviewers, clinician-administered checklists, or a medical record review. Across cohorts, participants with depression were excluded if they also reported a comorbid diagnosis of bipolar disorder, schizophrenia, or personality disorder as suggested in previous studies (Smith et al., 2013). In addition, controls in most samples were determined based on a negative response to the self-report questions used in the UK Biobank and 23andMe cohort or they were screened for the absence of lifetime major depressive disorder and randomly selected from the population (Howard et al., 2019;Wray et al., 2018).

Further details for these GWAS summary statistics are available in their corresponding publication (Howard et al., 2019). Data from the PGC were retrieved from a publicly available repository (https://pgc.unc.edu/for-researchers/download-results/). Data from 23andMe were obtained through the corresponding application procedure via a Data Transfer Agreement (https://research.23andme.com/dataset-access/).

### 2.3 Intracranial and subcortical brain volumes GWAS data

GWAS summary data for intracranial volume and nine subcortical brain volumes, namely the amygdala, brainstem, caudate nucleus, hippocampus, nucleus accumbens, pallidum, putamen, thalamus, and ventral diencephalon, were derived from a GWAS meta-analysis using data from four international sources. Briefly, the meta-analysis included up to 73,436 individuals of European ancestry from The Adolescent Brain Cognitive Development (ABCD) (Feldstein Ewing et al., 2018) and UK Biobank (Elliott et al., 2018) cohorts and the CHARGE (Thompson, 2015) and ENIGMA (Feldstein Ewing et al., 2018;Medland et al., 2022) consortia. As per ENIGMA’s protocols (Hibar et al., 2015;Satizabal et al., 2019), intracranial and subcortical brain volumes were defined as the mean volume of the left and right brain hemispheres, except for the brainstem, for which the total volume was used. All brain volumes were measured in cubic centimeters.

All cohorts leveraged the publicly available FreeSurfer package tool (https://surfer.nmr.mgh.harvard.edu/) to process the MRI scans of participants (Fischl, 2012;Fischl et al., 2002). For subcortical brain volumes, FreeSurfer leverages its own segmentation tool “recon-all,” which combines probabilistic atlas information, key anatomical features, and intensity from the MRI data to perform the segmentation (Fischl, 2012;Fischl et al., 2002). The definition of the borders of brain regions and structures relies on its own built-in atlases. These brain atlases are manually curated by the FreeSurfer team by leveraging data from thousands of subjects, which is being continuously refined and improved by the FreeSurfer team. Details regarding border definition for specific brain structures are available on the wiki (https://surfer.nmr.mgh.harvard.edu/fswiki/FreeSurferWiki). Furthermore, the processing pipeline for T1-weighted MRI images in FreeSurfer involves several steps including (i) Motion correction to align the volumes in space, (ii) Skull stripping to remove non-brain tissue from the MRI images, (iii) Intensity normalisation techniques to ensure consistent intensity values across MRI images, (iv) Tissue segmentation by classifying voxels in the brain into different tissue types, (v) Subcortical structure segmentation based on probabilistic atlases, key anatomical features, and intensity information specific to each subcortical structure, (vi) Surface reconstruction to enable visualisation and analysis of surface-based morphometry, (vii) Cortical parcellation based on gyral and sulcal patterns, and (viii) Registration of MRI images to a standard anatomical space (i.e., the Montreal Neurological Institute template) to enable group analysis and comparison of subjects (Fischl, 2012;Fischl et al., 2002).

GWAS data from the ENIGMA and CHARGE consortia were obtained via a data application. GWAS for the ABCD and UK Biobanks cohorts were conducted using a linear mixed model accounting for genotyping array, sex, age, sex*age, age^2^, sex*age^2^, and the first 20 genetic principal components using BOLT-LMM (Loh et al., 2015). GWAS for the volume of subcortical brain structures were adjusted for the effects of total intracranial volume (ICV) to control for potential variation in subcortical brain volumes due to differences in head size among participants (Hedman et al., 2011). Variants with a low minor allele frequency (<0.01) or a low-quality imputation score (<0.60) were excluded from the analysis. On average, GWAS summary statistics for each brain volume include a total of 5,710,757 genetic variants.

Both sex and age are known to influence intracranial and subcortical brain volumes (Armstrong et al., 2019;Dima et al., 2022;Ziegler et al., 2012). For instance, men are known to have, on average, larger brains than women and divergence in brain development between sexes has been noted during childhood, adolescence, and early adulthood. In addition, intracranial and subcortical brain volumes fluctuate throughout the lifespan in a non-linear manner, substantially increasing early in development, but slowly declining as individuals age, particularly in the 5th and 6th decades of life, reflecting a quadratic dependency. The samples in the present study include both males and females and participants from different age ranges, from children and teenagers in the ABCD cohort to older adults from the UKBB cohort. Thus, the GWAS summary statistics included age-squared, and the interaction between sex and age-squared on intracranial and subcortical brain volumes to account for brain volume variation across the lifespan given that the samples under study included participants of different age ranges and differences in neurodevelopmental trajectories that have been noted between males and females. In addition, the summary statistics included the interaction between sex and age as a representation of the linear term required in the quadratic model, which includes the interaction between sex and age-squared. In the quadratic model, the linear factor, sex*age influences a shift in symmetry away from the y-axis, given that it is unlikely that individuals reach their brain volume plateau exactly halfway through their lifespan (usually this happens at around 25 years of age). Data from all cohorts, including ENGIMA and CHARGE, were controlled for age and sex. Full details on genotyping, protocols for data acquisition, and phenotype definition are available in the corresponding publications (Satizabal et al., 2019;Thompson et al., 2014,^2020^).

### 2.4 Genetic correlations

We examined the pairwise genetic overlap between depression and 10 brain volumes using the linkage-disequilibrium score regression (LDSC) method (Bulik-Sullivan et al., 2015), which is publicly available and implemented in Python (https://github.com/bulik/ldsc/blob/master/ldscore/ldscore.py). We accounted for multiple testing using a traditional Bonferroni multiple testing correction. Therefore, we set a significance threshold of*P*-value < 0.05 / 10 = 5 x 10^-3^. Briefly, LDSC estimates the amount of genetic variation tagged by each SNP due to linkage disequilibrium, and leverages GWAS summary statistics for two phenotypes to perform a bivariate regression of the product of the z-scores from the two GWAS on the LD scores. The slope of the regression line is an estimate of the genetic correlation between the two traits, indicating the proportion of shared genetic influence (Bulik-Sullivan et al., 2015).

### 2.5 Pairwise GWAS

We leveraged the pairwise GWAS (GWAS-PW) method (Pickrell et al., 2016) to delineate the genetic overlap between depression and 10 brain volumes. Analyses were performed for depression and each of the 10 brain volumes separately. Briefly, this method splits GWAS summary statistics into 1,703 independent genomic segments based on linkage disequilibrium patterns (Pickrell et al., 2016). Later on, for each segment, the posterior probability of the association (PPA) is estimated to assess the likelihood of an observed association between genetic variants and a specific phenotype given the observed genotypic data (Pickrell et al., 2016). In GWAS-PW, PPA is estimated for four different models, including (i) the segment is uniquely associated with depression, (ii) the segment is uniquely associated with brain volume, (iii) the segment is associated with both depression and the brain volume through the same genetic variants, and (iv) the segment is associated with both depression and the brain volume but via different genetic variants. In the present study, we selected segments of the genome where model three (shared causal variants) had a PPA > 0.5 (García-Marín et al., 2023;Mitchell et al., 2020;Reyes-Pérez et al., 2024).

### 2.6 Functional annotation

We conducted functional annotation analyses across the genome by uploading our GWAS summary statistics to the FUMA online platform (Watanabe et al., 2017), which provides tools for annotating, interpreting, and visualising the results of GWAS (Watanabe et al., 2017). For each brain volume GWAS, we mapped lead SNPs for each locus to genes using MAGMA (de Leeuw et al., 2015) (v1.08). Briefly, MAGMA gene-based association analyses leverage GWAS summary statistics to map genetic variants (SNPs) to genes and aggregate SNP-level*P*-values to gene-level*P*-values, considering the linkage disequilibrium between SNPs (de Leeuw et al., 2015). We extracted functional annotation data for the exact genomic segments identified via the GWAS-PW method and selected significant genes after applying a Bonferroni multiple testing correction defined as*P *= 0.05 / [total number of genes for each subcortical brain structure].*P*-values reported in the results section correspond to the MAGMA output.

### 2.7 Gene expression

We performed MAGMA Tissue Expression Analysis to identify the tissues in which genes associated with depression are preferentially expressed. We leveraged gene expression data from the Genotype-Tissue Expression (GTEx) project (The GTEx Consortium*, 2013), which provides comprehensive gene expression profiles across a wide range of human tissues, including subcortical brain tissue for the hypothalamus, amygdala, nucleus accumbens, hippocampus, caudate nucleus, putamen, and substantia nigra. MAGMA uses a linear regression model to test the association between gene-based associations from the depression GWAS and the expression levels of these genes in different tissues. We corrected for multiple testing using a Bonferroni correction.*P*= 0.05 / [total number of genes for each subcortical brain structure].*P*-values reported correspond to the MAGMA output.

### 2.8 Local analysis of covariant annotation (LAVA)

Segments of the genome identified via GWAS-PW that also showed genes associated with depression and at least one brain volume were assessed using LAVA to further investigate local genetic correlations (Werme et al., 2022). Briefly, LAVA provides insights into how genetic variants in specific regions of the genome contribute to the genetic correlation between traits and is implemented in a software package written in R (Werme et al., 2022). In these methods, the genome is divided into independent LD blocks, ensuring that genetic variants within a block are highly correlated with each other but not with variants in other blocks (Werme et al., 2022). Then, for each LD block, LAVA estimates the local genetic correlation between the traits by computing the covariance between the effect sizes of SNPs for the traits within the block (Werme et al., 2022).

### 2.9 Phenotypic correlations

We complemented our genetic analyses by estimating phenotypic correlations (Pearson’s correlations) of depression and intracranial and subcortical brain volumes using data from the UK Biobank. We leveraged phenotypic data for 36,095 individuals of European ancestry with data for depression and intracranial and subcortical brain volumes. As for the GWAS analyses, intracranial and subcortical brain volumes were defined as the mean volume of the left and right brain hemispheres, except for the brainstem, for which the total volume was used. All brain volumes were measured in cubic centimeters. We used two definitions of depression available in the UKB: Field code 2090*“Have you ever seen a general practicioner for nerves, anxiety, tension or depression?”,*and field code 2100 “*Have you ever seen a psychiatrist for nerves, anxiety, tension or depression?”.*We provide results for each item in the Supplementary Materials. The main text shows results for the combination of these items, where a patient was considered to have depression if responding “Yes” to any of the UK Biobank field codes (2090 and 2100). We accounted for multiple testing using a traditional Bonferroni multiple testing correction. Therefore, we set a significance threshold of*P*-value < 0.05 / 10 = 5 x 10^-3^.

### 2.10 Sensitivity analyses

In light of the complexity and the well-established genetic overlap across neuropsychiatric disorders, we performed, as sensitivity analyses, GWAS-PW analyses for several neuropsychiatric disorders, including bipolar disorder (Mullins et al., 2021) and schizophrenia (Trubetskoy et al., 2022) with intracranial and subcortical brain volumes. These analyses, as performed with depression, were followed by MAGMA analyses. We corrected for multiple testing for bipolar disorder and schizophrenia in the same way we did for the analyses performed for depression. These sensitivity analyses enabled us to assess if genes were specifically associated with depression and brain morphometry or if the identified genes could be potentially related to processes that are affected across correlated mental disorders. Full details for the GWAS summary statistics for bipolar disorder and schizophrenia are available in their corresponding publications. Briefly, for both of these phenotypes, summary statistics include adult participants from European ancestry. Bipolar disorder dataset included 41,917 cases and 371,579 controls (Mullins et al., 2021), while the dataset for schizophrenia included 53,386 cases and 77,258 controls (Trubetskoy et al., 2022).

## 3 Results

### 3.1 Genetic correlations

**Fig. 1.**
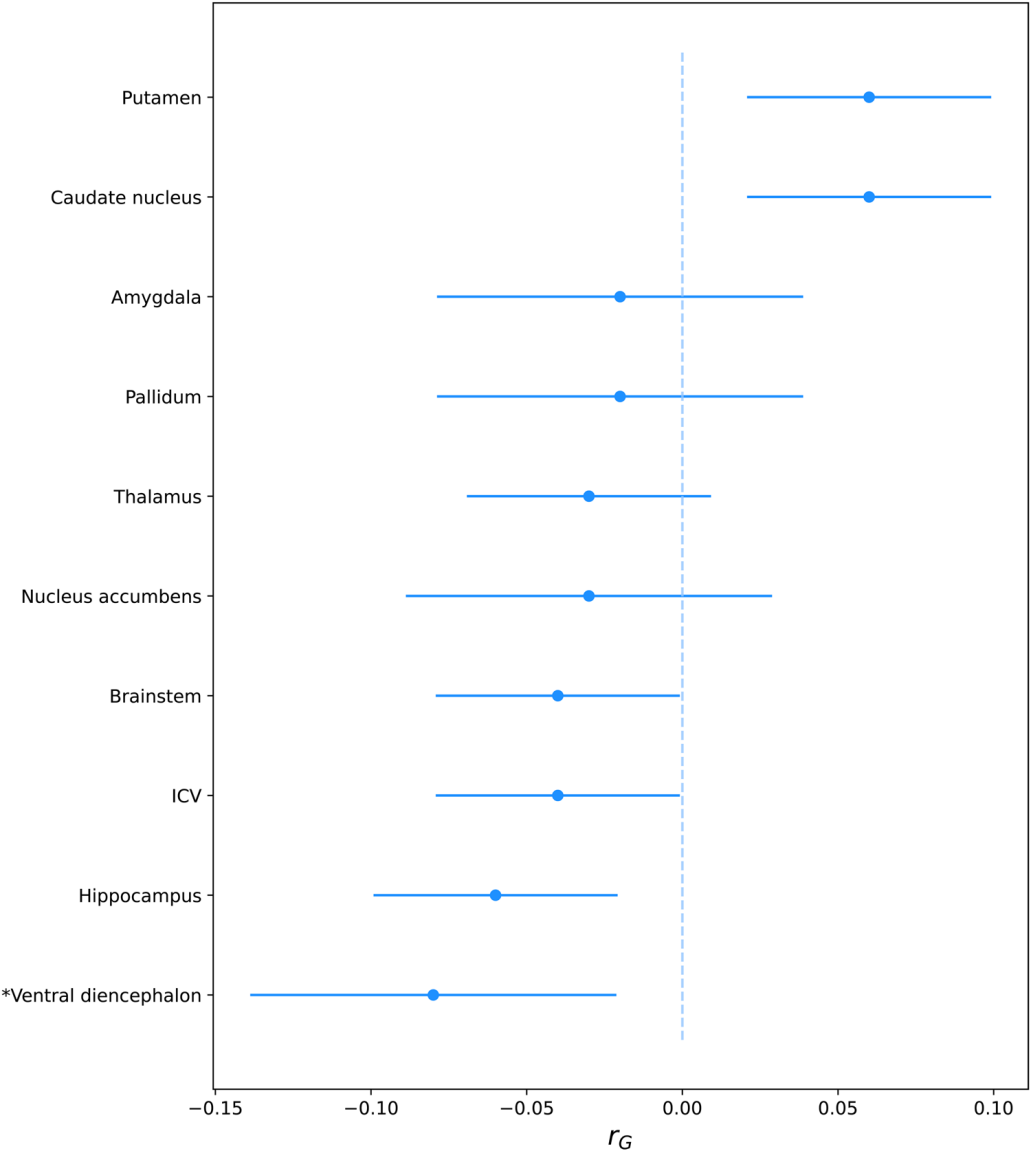
Linkage disequilibrium score regression (LDSC) estimates of the genetic correlation (rG) with 95% confidence intervals between depression with intracranial and subcortical brain volumes. Statistically significant genetic correlations after multiple testing correction (i.e.,*P*< 0.05 / 10 [number of brain volumes under study] = 0.005) are marked with an asterisk (*). ICV = Intracranial volume.

We estimated pairwise genetic correlations at the whole-genome level between depression and intracranial and subcortical brain volumes using LD score regression (Fig. 1;Table 1). The negative genetic correlation between depression and ventral diencephalon volume (rG = -0.08,*P*= 1.40 x 10^-3^) was the only one to survive multiple testing corrections. In addition, we identified a nominally significant negative genetic correlation (*P*< 0.05) between depression and the volume of the hippocampus (rG = -0.06,*P*= 2.00 x 10^-2^). Conversely, we observed positive genetic correlations of nominal significance between depression and the volumes of the putamen (rG = 0.06,*P*= 3.90 x 10^-2^) and the caudate nucleus (rG = 0.06,*P*= 1.28 x 10^-2^).

**Table 1.** Linkage disequilibrium score regression (LDSC) estimates of the genetic correlation (rG) of depression with intracranial and subcortical brain volumes.

Brain volume	rG	SE	*P*
*Ventral diencephalon	-0.08	0.03	1.40E-03
Caudate nucleus	0.06	0.02	1.28E-02
Hippocampus	-0.06	0.02	2.00E-02
Putamen	0.06	0.02	3.90E-02
ICV	-0.04	0.02	9.35E-02
Brainstem	-0.04	0.02	6.46E-02
Nucleus accumbens	-0.03	0.03	2.92E-01
Thalamus	-0.03	0.02	3.62E-01
Pallidum	-0.02	0.03	4.08E-01
Amygdala	-0.02	0.03	5.72E-01

Statistically significant genetic correlations after multiple testing correction (i.e.,*P*< 0.05 / 10 = 5 x 10^-3^) are marked with an asterisk (*). ICV = Intracranial volume.

### 3.2 GWAS-pairwise, functional annotation, tissue expression, and LAVA

**Fig. 2.**
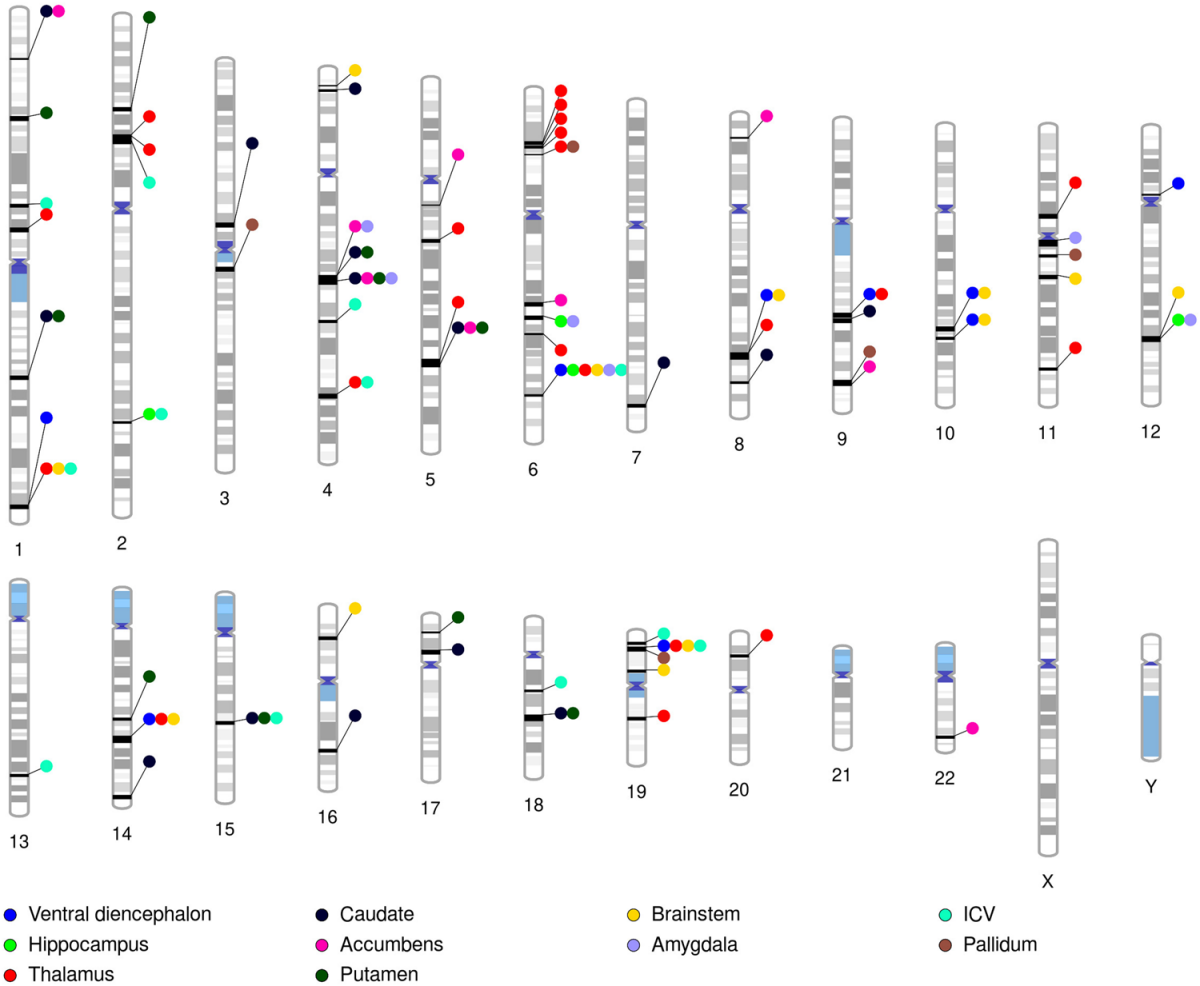
Ideogram highlighting genomic segments influencing the aetiology of depression and subcortical brain volumes via the same genetic variants. Genomic segments influencing depression and intracranial volume (ICV) are also shown.

We further investigated the genetic relationship of depression with intracranial and subcortical brain structures at the local genetic level using the GWAS-pairwise method. With this approach, we observed 104 genome segments influencing depression and the volume of at least one brain structure via the same genetic variants (Fig. 2). For brain volumes with shared segments of the genome with depression, we mapped genetic variants in the corresponding genomic segments to protein-coding genes. All brain volumes, except the pallidum, displayed associated genes after Bonferroni multiple testing correction (Supplementary Materials). We observed 96 genes associated with depression and at least one brain volume, of which 67 were unique.

Putamen and brainstem volume shared the association of 32 and 19 genes with depression, respectively. These were followed by hippocampal and intracranial volumes, with 16 and 12 associated genes with depression. Furthermore, the volume of the ventral diencephalon shared eight genes, and the caudate nucleus five. The brain volumes with the fewest common associated genes with depression were the amygdala (2 genes), thalamus (1 gene), and nucleus accumbens (1 gene).

We observed that some genes were associated with depression and multiple brain volumes. In particular, we observed that genes associated with depression also tend to be associated with three different groups: (i) the volume of the brainstem, hippocampus, and ventral diencephalon; (ii) intracranial and hippocampus volume; and (iii) ICV and the volume of the brainstem and ventral diencephalon. For instance, glycosylated integral membrane protein 1 (*GINM1*), katanin catalytic subunit A1 (*KATNA1*), large tumour suppressor kinase 1 (*LATS1*), nucleoporin 43 (*NUP43*), pericentriolar material 1 (*PCMT1*), and LDL receptor-related protein 11 (*LRP11*) were associated with depression and the volumes of the brainstem, hippocampus, and ventral diencephalon.

Ten genes were associated with depression, ICV, and the volume of the hippocampus, among them Heat Shock Protein Family E (Hsp10) Member 1 (*HSPE1*), MOB Family Member 4, Phocein (*MOB4*), and Coenzyme Q10B (*COQ10B*). In addition, the insulin receptor (*INSR*) gene was associated with ICV and the volumes of the brainstem and the ventral diencephalon, as was AKT serine/threonine kinase 3 (*AKT3*), which was also associated with the volume of the thalamus.

With tissue expression analyses using the GWAS summary statistics for depression, we observed that the subcortical brain tissues where associated genes are highly expressed include the hypothalamus, amygdala, nucleus accumbens, hippocampus, caudate nucleus, putamen, and substantia nigra (Supplementary Materials).

Based on our GWAS-PW results, we used LAVA to further explore the local genetic correlations in genomic segments with associated genes with depression and at least one brain volume (Supplementary Materials). Results remained largely consistent, with strong (|rho| > 0.80) local genetic correlations observed between depression and brain volumes.

### 3.3 Phenotypic correlations

We estimated phenotypic correlations using data from the UK Biobank and observed predominantly statistically significant negative correlations between depression and intracranial and all subcortical brain volumes (Supplementary Materials). ICV (r = -0.09,*P*= 4.06 x 10^-70^) and the volume of the ventral diencephalon (r = -0.08*P*= 6.12 x 10^-61^) showed the largest genetic correlations with depression, while the smallest were observed for the volume of the nucleus accumbens (r = -0.03,*P*= 5.99 x 10^-08^) and caudate nucleus (r = -0.03,*P*= 2.52 x 10^-10^).

### 3.4 Sensitivity analyses

We performed LDSC, GWAS-PW, and MAGMA analyses of bipolar disorder and schizophrenia with intracranial and subcortical brain volumes to compare results with those obtained for depression and see whether the reported genes for depression were also associated with other psychiatric disorders.

We did not observe significant genetic correlations between bipolar disorder and intracranial and subcortical brain volumes using LDSC after correcting for multiple testing (Supplementary Materials). Nonetheless, we observed negative genetic relationships of nominal significance (*P*< 0.05) with the pallidum (rG = -0.07,*P*= 2.25 x 10^-2^), the ventral diencephalon (rG = -0.07,*P*= 3.27 x 10^-2^), and the brainstem (rG = -0.05,*P*= 4.34 x 10^-2^).

For schizophrenia, we observed a significant genetic correlation after multiple testing correction with the volume of the ventral diencephalon (rG = -0.08,*P*= 3.00 x 10^-3^), followed by negative genetic relationships of nominal significance (*P*< 0.05) with the volumes of the pallidum (rG = -0.07,*P*= 5.50 x 10^-3^), thalamus (rG = -0.07,*P*= 1.44 x 10^-2^), hippocampus (rG = -0.06,*P*= 2.93 x 10^-2^), and brainstem (rG = -0.05,*P*= 4.65 x 10^-2^).

GWAS-PW analyses uncovered 206 genomic segments with genetic variants influencing schizophrenia and at least the volume of one brain volume, while for bipolar disorder we identified 67 segments of the genome (Supplementary Materials). Among these segments of the genome, we observed 143 gene-based associations with schizophrenia and at least one brain volume, and 51 gene-based associations with bipolar disorder and at least one brain volume. These gene associations involved 82 and 36 unique genes for schizophrenia and bipolar disorder, respectively.

Comparing findings for depression, schizophrenia, and bipolar disorder, we observed that 13 unique genes were associated with depression and at least one brain volume. These 13 genes were also associated with schizophrenia or bipolar disorder and at least one brain volume. In contrast, out of the genes associated with depression and at least one brain volume, 54 were specific for depression findings, meaning that most of the genes identified for depression and brain volumes were not identified when performing the analyses for schizophrenia and bipolar disorder (Supplementary Materials).

Genes that were associated with brain volumes, depression, and schizophrenia or bipolar disorder included*AC011997.1*,*AKT3*,*BANK1*,*BOLL*,*DCC*,*HSPD1*,*HSPE1*,*HSPE1-MOB4*,*MOB4*,*PLCL1*,*RFTN2*,*SF3B1*, and*TRPS1*(Supplementary Materials). No gene was associated with brain volumes and all three neuropsychiatric disorders (depression, bipolar disorder, and schizophrenia).

## 4 Discussion

In the present study, we investigated the genetic associations of depression with intracranial and nine subcortical brain volumes. We present genome-wide genetic findings for the first time for the ventral diencephalon and depression in individuals of European ancestry using GWAS summary statistics. One of the primary significant findings of the present study was the negative genetic correlation, at the whole-genome level, between depression and the volume of the ventral diencephalon. We observed a negative genetic correlation, that did not survive multiple testing correction, between depression and hippocampal volume, while positive genetic correlations that did not survive multiple testing correction were observed with the volume of the putamen and the caudate nucleus. We further investigated the genetic associations between depression and intracranial and subcortical brain volumes and uncovered 104 genomic segments influencing the aetiology of depression and at least one brain volume. Interrogation of these genomic segments using LAVA suggests that there may be various locus involved in correlations observed between depression and brain volumes.

Our genetic correlations findings at the whole-genome level are for the most part consistent with those reported in previous GWAS studies (Ohi et al., 2020). Nonetheless, a previous study leveraging genetic data for Mexican Americans conducted a multipoint quantitative-trait linkage analysis in general pedigrees and identified larger genetic correlations between subcortical volumes and depression (Glahn et al., 2012). We attribute these differences in the magnitude of the genetic correlations between studies to differences in the ancestry of participants, sample sizes, and methodologies implemented in the studies. Nonetheless, we note that both studies show predominantly negative genetic correlations between depression and subcortical brain volumes.

Previous research has highlighted the role of the hippocampus in the aetiology of depression (Campbell & MacQueen, 2004;MacQueen & Frodl, 2010;Roddy et al., 2019;Sheline, 2011). Most observational studies suggest that individuals with depression tend to have smaller hippocampal volumes, with those showing recurrent or chronic depression having a more pronounced volume reduction (Malykhin et al., 2010;Sheline, 2011;Tartt et al., 2022). However, genetic studies have reported positive genetic correlations (*P*= 0.02) between depression and the volume of the hippocampus (Wigmore et al., 2017). In the present study, we observed a nominally significant (*P*< 0.05) negative genetic correlation at the whole-genome level between depression and the hippocampus volume, and we observed multiple genes associated with both phenotypes at the local genetic level. We attribute the inconsistency of the correlation at the whole-genome level to differences in sample sizes between previous studies and the present one.

The importance of functional annotation analysis relies on its potential to (i) provide biological context to GWAS findings, helping to understand how genetic variants influence traits and (ii) annotation can help prioritise genes and pathways as potential drug targets for therapeutic intervention. The comprehensive analysis of GWAS results, linking genetic associations to functional annotations and biological pathways, contributes to advance our understanding of the genetic basis of complex phenotypes.

Notably, in the present study, known GWAS top genes for depression were associated with specific brain volumes. For instance, DCC netrin 1 Receptor (*DCC*) was associated with the volume of the putamen (*P*= 2.49 x 10^-40^) and the caudate nucleus (*P*= 1.53 x 10^-11^). Similarly, apoptosis-resistant E3 ubiquitin protein ligase 1 (*AREL1*;*P*= 7.57 x 10^-11^), dihydrolipoamide S-Succinyltransferase (*DLST*;*P*= 7.57 x 10^-11^), FCF1 rRNA-processing protein (*FCF1*;*P*= 1.66 x 10^-10^), YLP motif containing 1 (*YLPM1*;*P*= 1.11 x 10^-10^), ribosomal protein S6 kinase-like 1 (*RPS6KL1*;*P*= 1.27 x 10^-10^), and prospero homeobox 2 (*PROX2*;*P*= 3.05 x 10^-10^) were associated with the volume of the brainstem. Overall, we observed that genes associated with depression were also associated with specific groups of brain volumes, including (i) brainstem, ventral diencephalon, and intracranial volume; (ii) brainstem, ventral diencephalon, and hippocampus volume; and (iii) intracranial and hippocampus volume. In addition, we identified several genes that were uniquely associated with depression and the volume of the putamen or the brainstem. Below, we discuss the biological relevance of some protein-coding genes associated with the genome segments influencing depression and at least one brain volume. These genes were predominantly involved in dopamine neurotransmission, mesocorticolimbic functional connectivity, GABAergic transmission, and the insulin signalling pathway.

The neurobiological understanding of depression has been frequently explained and popularised by the monoamine-deficiency hypothesis (Delgado, 2000;Hasler, 2010). This hypothesis states that depression is most likely explained by depletion, to different extents, of the three main monoamine neurotransmitters: serotonin, norepinephrine, and dopamine. This depletion is mostly observed in the central nervous system, particularly in the midbrain and brainstem nuclei, with projections to other areas of the human brain (Hasler, 2010). Historically, serotonin and norepinephrine have been the most widely studied neurotransmitters in depression (Hasler, 2010). Nonetheless, it has been observed that dopamine is another monoamine molecule influencing the aetiology of depression, with some studies suggesting that depression could arise from neurodevelopmental maladaptations in dopamine-regulated processes early in development (Delva & Stanwood, 2021).

Dopaminergic neurons are predominantly found in the midbrain, particularly in the ventral tegmental area, the substantia nigra, and the hypothalamus. Furthermore, four principal dopaminergic pathways have been identified in the human brain. These include the mesolimbic pathway (going from the ventral tegmental area to the nucleus accumbens), the mesocortical pathway (going from the ventral tegmental area to the prefrontal cortex), the nigrostriatal pathway (going from the substantia nigra to the caudate nucleus), and the tuberoinfundibular pathway (going from the tuberal hypothalamus to the pituitary gland) (C. Li et al., 2019).

Both humans and rodents share similar features in their homologous mesocorticolimbic dopamine systems. Therefore, rodent studies have been a primary approach to understanding the dopaminergic system in the human brain. Specifically, dopaminergic signals project from the brainstem to the dorsal striatum and further into multiple cortical and subcortical regions of the limbic system (Vosberg et al., 2020). In addition, it has been observed that the density of mesocortical dopamine fibers increases early in development, particularly from adolescence to adulthood (Vosberg et al., 2020).

It has been reported that genetic variants in the*DCC*gene are associated with depression (Leday et al., 2018;Vosberg et al., 2020) and total brain volume (Morgunova et al., 2021). Furthermore, some studies have suggested*DCC*as a potential antidepressant target (H.-J. Li et al., 2020;Torres-Berrío et al., 2020). In particular, it has been hypothesised that Netrin-1 signaling disruptions could be mediated by*DCC*, ultimately contributing to disturbances in dopamine neurotransmission and mesocorticolimbic functional connectivity (Furman et al., 2011;Russo & Nestler, 2013;Vosberg et al., 2020). Netrin-1 is an axon guidance protein that facilitates the development of axons towards their respective targets. Notably, DCC is commonly known as a cell adhesion molecule that is capable of mediating the influence of Netrin-1 on the outgrowth of axons. However, the association between*DCC*and depression has been mostly explained by observations in cortical structures and, to a lesser extent, with subcortical brain volumes, particularly for the volume of the putamen (Hibar et al., 2015). For instance, some studies have reported high*DCC*mRNA levels in the prefrontal cortex of antidepressant-free depression patients (Manitt et al., 2013;Torres-Berrío et al., 2017). In the present study, we provide further evidence confirming the association of*DCC*with depression and the putamen volume and suggest potential links to the volume of the caudate nucleus. We speculate that these associations could be explained by the mediating role of*DCC*, which is known to be highly expressed early in neurodevelopment (Kang et al., 2011), in Netrin-1 function in the human subcortical brain, resulting in connectivity abnormalities, particularly in the mesolimbic and mesocortical pathways.

In the present study, we observed that the*INSR*gene was associated with depression and intracranial, brainstem, and ventral diencephalon volumes. Specifically,*INSR*plays a key role in activating the insulin signaling pathway, and*INSR*dysfunction is well known to lead to insulin resistance, which, in turn, may lead to hyperglycemia (Ariza Jiménez et al., 2019;Mesika et al., 2018;Payankaulam et al., 2019). Similarly, we identified that the genes*HSPE1*and*HSPD1*were associated with depression, ICV, and the hippocampus volume. In particular,*HSPE1*and*HSPD1*encode heat shock proteins, which have protective effects reducing the likelihood of insulin resistance (Moin et al., 2020;Zilaee & Shirali, 2016). However, in the presence of hyperglycemia, most heat shock proteins reduce their expression levels (Moin et al., 2020;Zilaee & Shirali, 2016). In addition, insulin resistance has been reported to affect the dopaminergic system (Fiory et al., 2019;Kleinridders & Pothos, 2019;Kleinridders et al., 2015;Schell et al., 2021;Stouffer et al., 2015) by potentially inhibiting dopaminergic neurons, reducing neural dopamine release and the inactivation of monoamine neurotransmitters (Caravaggio et al., 2015). Our findings complement those of previous studies that have sought to describe the potential role of insulin regulation in the aetiology of depression (de M. Lyra e Silva et al., 2019;Kleinridders et al., 2015;Zou et al., 2020). For instance, observational studies have suggested that insulin resistance could increase the risk of depression (Zou et al., 2020) due to dysglycemia (Gilsanz et al., 2018), while genetic studies have not been able to fully delineate putative causal genetic effects (Frangou et al., 2019) or the underlying biological mechanisms of this association (de M. Lyra e Silva et al., 2019;Kleinridders et al., 2015). Overall, our results suggest that the association between depression, ICV, and the volumes of subcortical brain structures could be explained by the dysfunction of the insulin signaling pathway, leading to a reduction of dopamine and monoamines levels (Kleinridders et al., 2015).

Although less prominent than the monoamine-deficiency hypothesis, the GABAergic Deficit Hypothesis has also been formulated in an effort to explain the aetiology of depression (Duman et al., 2019;Luscher & Fuchs, 2015;Luscher et al., 2011). For instance, studies have observed that depressed individuals exhibit a reduction in brain levels of the inhibitory neurotransmitter γ-aminobutyric acid (GABA) along with abnormalities in the composition of other principal receptors, commonly referred to as GABAA receptors, which regulate GABA inhibitory activity (Luscher et al., 2011). Therefore, the GABAergic Deficit Hypothesis states that abnormalities in GABAergic transmission play an essential role in the etiological sequelae of depression, and it has been suggested to be reversed by the effects of monoaminergic antidepressants (Luscher et al., 2011).

GABAergic transmission is particularly relevant in the hippocampus as it profoundly influences hippocampal neurogenesis and neural maturation, which, in turn, are well-established substrates of antidepressant treatments (Luscher et al., 2011). Similarly to the study of dopaminergic pathways in depression, GABA deficits have mostly been reported on plasma, cortical tissue, or cerebrospinal fluid (Bhagwagar et al., 2008;Luscher et al., 2011) but not in subcortical brain structures. In the present study, ICV and hippocampal volume were associated with*PLCL1*, which, in turn, is known to have a crucial role in the development of glutamatergic and GABAergic synapses (Devor et al., 2017;Zheng et al., 2021), specifically in the hippocampus. In addition, the hippocampus is highly susceptible to the effect of increased levels of corticotropin-releasing factor and cortisol in response to chronic stress. These chronic stress markers over-activate the hypothalamic-pituitary-adrenal axis, which may ultimately result in hypoactivity of the hippocampus and decreased neurotransmission (Tafet & Nemeroff, 2016). Our findings suggest that the relationship between depression and the hippocampus could be explained by the role of*PLCL1*in GABAergic transmission abnormalities, influencing brain development and normal neural function, perhaps as a result of exposure to chronic environmental or emotional stressors.

Our sensitivity analyses highlight the potential role of the ventral diencephalon in the aetiology of depression, bipolar disorder, and schizophrenia. Furthermore, we observed that most of the genes identified for depression and at least one brain volume are specific for depression and were not observed for the relationships between bipolar disorder or schizophrenia with brain volumes. These findings suggest that although there may be common brain processes influencing, to different extents, the aetiology of multiple neuropsychiatric disorders, there are several processes that are likely to be affected specifically in each neuropsychiatric disorder.

Overall, we provide evidence for genetic factors underlying the association of depression with intracranial and subcortical brain volumes. We note that depression is a highly complex mental health disorder with a multifaceted aetiology, which is likely driven by several different biological pathways and environmental stressors that are also likely to interact with each other. Thus, we encourage future studies to seek to fill in the gap of knowledge for the biological theories describing the onset and recurrence of depression in a neurobiological context. Strengths of the present study include the use of a larger sample size than the one used in previous studies seeking to estimate the genetic overlap between depression and specific brain volumes (Mathias et al., 2016;Satizabal et al., 2019;Wigmore et al., 2017), which ultimately results in robust statistical power to perform genetic association analyses.

Limitations of the present study must be acknowledged. For instance, GWAS summary data used in the present study are only representative of individuals of European ancestry. Therefore, the generalisability of results presented here must be cautiously addressed until replicated in samples from different ancestral backgrounds. We note that the GWAS summary statistics for brain volumes do not formally exclude participants with depression or other neuropsychiatric disorders. Nonetheless, samples included in these GWASs do not necessarily come from cohorts enriched for participants with neuropsychiatric disorders. We were unable to perform LAVA for some genomic segments identified via GWAS-PW given that only a few SNPs were available within a given LD block. These are marked as NA in the Supplementary Materials.

GWAS summary statistics for depression leveraged for the present study are based on a broad definition of lifetime history of depression that combines self-reported diagnoses with structured diagnostic interviews. As mentioned previously, depression is a complex psychiatric disorder and it is crucial that future studies aim to distinguish between structured diagnostic interviews, self-reported symptoms, and treatments, as these elements vary in their representation of depression’s severity and duration. Moreover, given that increasing discovery sample sizes lead to the identification of more associations (Hong & Park, 2012), we highlight that null findings here do not necessarily indicate the absence of an association. Lastly, the imaging and visualisation analysis was performed with the FreeSurfer package tool, which includes the superior cerebellar peduncle as part of the brainstem. We note that the superior cerebellar peduncle connects the cerebellum to the brainstem (Unverdi & Alsayouri, 2023); nonetheless, the cerebellar peduncle is not a potential anatomical structure of the brainstem. This is a limitation of the segmentation conducted by the FreeSurfer package tool.

## 5 Conclusions

In summary, we leveraged genome-wide data to investigate the genetic overlap of depression with intracranial and subcortical brain volumes. Our results suggest that genetic variants that influence intracranial and subcortical brain volumes are located within genes expressed in brain tissue whose dysfunction may contribute to the development of depression. Specifically, we provide evidence for molecular pathways involving dopamine neurotransmission, mesocorticolimbic functional connectivity, GABAergic transmission, and the insulin signalling pathway. Despite the intricate neurobiology involved in the pathophysiology of depression, our findings contribute to advancing our understanding of the genetic basis of the relationship between depression and the human brain, which, in turn, has the potential to open novel research avenues and aid the development of prevention and treatment strategies.

## Data and Code Availability

Full summary-level data for depression, including the 23andMe cohort, are available upon request through a Data Transfer Agreement and the appropriate application procedure (https://research.23andme.com/dataset-access/). No custom code was used in this study. Publicly available software tools were used to perform genetic analyses and are referenced throughout the manuscript.

## Author Contributions

L.M.G.M. and M.E.R. conceived and directed the study. L.M.G.M. performed the statistical and bioinformatic analysis with support and input from N.G.M., M.E.R., N.S.O., L.K.M.H., B.L.M., L.S., and M.M.M. L.M.G.M. wrote the first draft of the paper and integrated input and feedback from all co-authors. All authors contributed to the interpretation of the results and provided feedback on the preliminary versions of the manuscript.

## Funding

L.M.G.M. is supported by a UQ Research Training Scholarship from The University of Queensland (UQ). M.E.R. thanks support from the Rebecca L Cooper Medical Research Foundation through an Al & Val Rosenstrauss Fellowship (F20231230). B.L.M. is supported by the Australian National Health and Medical Research Council Investigator grant scheme (APP2017176).

## Declaration of Competing Interest

The authors declare no competing interests.

## Acknowledgments

We would like to thank the research participants and employees of 23andMe, Inc. for making the present work possible.

## Supplementary Material

The Supplementary Material section includes additional tables that enhance the understanding of the findings of the present study.Supplementary Table 1shows the sample size contribution per cohort for brain volumes GWAS.Supplementary Tables 2 to 10present the statistically significant MAGMA results for various brain structures, including the Accumbens, Thalamus, Putamen, Caudate Nucleus, Brainstem, Intracranial Volume, Ventral Diencephalon, Amygdala, and Hippocampus.Supplementary Tables 11, 12, and 13show results for tissue expression, LAVA, and phenotypic correlation analyses.Supplementary Tables 14 and 15show LDSC regression results for bipolar disorder and schizophrenia with brain volumes, respectively. Lastly,Supplementary Table 16provides the lists of genes associated with at least one brain volume, depression, and bipolar disorder or schizophrenia, whileSupplementary Table 17shows the list of genes uniquely associated with at least one brain volume and depression. Supplementary material for this article is available with the online version here:https://doi.org/10.1162/imag_a_00291

## Supplementary Material

Supplementary Material

## References

[b1] Aman , A. M. , García-Marín , L. M. , Thorp , J. G. , Campos , A. I. , Cuellar-Partida , G. , Martin , N. G. , & Rentería , M. E. ( 2022 ). Phenome-wide screening of the putative causal determinants of depression using genetic data . Human Molecular Genetics , 31 ( 17 ), 2887 – 2898 . 10.1093/hmg/ddac081 35394011

[b2] Ariza Jiménez , A. B. , López Siguero , J. P. , Martínez Aedo Ollero , M. J. , Del Pino de la Fuente , A. , Isabel, & Gea , L. ( 2019 ). INSR gene mutation. Insulin resistance with low prevalence in pediatrics. A case review . Endocrinología Diabetes Y Nutrición (English Ed) , 66 ( 9 ), 588 – 589 . 10.1016/j.endien.2019.04.004 31229400

[b3] Armstrong , N. M. , An , Y. , Beason-Held , L. , Doshi , J. , Erus , G. , Ferrucci , L. , Davatzikos , C. , & Resnick , S. M. ( 2019 ). Sex differences in brain aging and predictors of neurodegeneration in cognitively healthy older adults . Neurobiology of Aging , 81 , 146 . 10.1016/j.neurobiolaging.2019.05.020 31280118 PMC9310670

[b4] Athira , K. V. , Bandopadhyay , S. , Samudrala , P. K. , Naidu , V. G. M. , Lahkar , M. , & Chakravarty , S. ( 2020 ). An overview of the heterogeneity of major depressive disorder: Current knowledge and future prospective . Current Neuropharmacology , 18 ( 3 ), 168 . 10.2174/1570159x17666191001142934 31573890 PMC7327947

[b6] Bhagwagar , Z. , Wylezinska , M. , Jezzard , P. , Evans , J. , Boorman , E. , Matthews P, M. , & Cowen P, J. ( 2008 ). Low GABA concentrations in occipital cortex and anterior cingulate cortex in medication-free, recovered depressed patients . The International Journal of Neuropsychopharmacology/Official Scientific Journal of the Collegium Internationale Neuropsychopharmacologicum , 11 ( 2 ), 255 – 260 . 10.1017/S1461145707007924 17625025

[b7] Bromet , E. , Andrade , L. H. , Hwang , I. , Sampson , N. A. , Alonso , J. , de Girolamo , G. , de Graaf , R. , Demyttenaere , K. , Hu , C. , Iwata , N. , Karam , A. N. , Kaur , J. , Kostyuchenko , S. , Lépine , J.-P. , Levinson , D. , Matschinger , H. , Mora , M. E. M. , Browne , M. O. , Posada-Villa , J. , … Kessler , R. C. ( 2011 ). Cross-national epidemiology of DSM-IV major depressive episode . BMC Medicine , 9 ( 1 ), 1 – 16 . 10.1186/1741-7015-9-90 21791035 PMC3163615

[b8] Bulik-Sullivan , B. K. , Loh , P.-R. , Finucane , H. K. , Ripke , S. , Yang , J. , Patterson , N. , Daly , M. J. , Price , A. L. , & Neale , B. M. ( 2015 ). LD Score regression distinguishes confounding from polygenicity in genome-wide association studies . Nature Genetics , 47 ( 3 ), 291 – 295 . 10.1038/ng.3211 25642630 PMC4495769

[b9] Campbell , S. , & MacQueen , G. ( 2004 ). The role of the hippocampus in the pathophysiology of major depression . Journal of Psychiatry & Neuroscience: JPN , 29 ( 6 ), 417 . 10.1093/acprof:oso/9780199592388.003.0015 15644983 PMC524959

[b10] Caravaggio , F. , Borlido , C. , Hahn , M. , Feng , Z. , Fervaha , G. , Gerretsen , P. , Nakajima , S. , Plitman , E. , Chung , J. K. , Iwata , Y. , Wilson , A. , Remington , G. , & Graff-Guerrero , A. ( 2015 ). Reduced insulin sensitivity is related to less endogenous dopamine at D2/3 receptors in the ventral striatum of healthy nonobese humans . The International Journal of Neuropsychopharmacology/Official Scientific Journal of the Collegium Internationale Neuropsychopharmacologicum , 18 ( 7 ), pyv014 . 10.1093/ijnp/pyv014 PMC4540108 25716779

[b11] Christensen , M. C. , Wong , C. M. J. , & Baune , B. T. ( 2020 ). Symptoms of major depressive disorder and their impact on psychosocial functioning in the different phases of the disease: Do the perspectives of patients and healthcare providers differ? Frontiers in Psychiatry/Frontiers Research Foundation , 11 , 280 . 10.3389/fpsyt.2020.00280 PMC7193105 32390877

[b12] de Leeuw , C. A. , Mooij , J. M. , & Heskes , T. ( 2015 ). MAGMA: Generalized gene-set analysis of GWAS data . PLoS Computational Biology , 11 ( 4 ), e1004219 . 10.1371/journal.pcbi.1004219 25885710 PMC4401657

[b13] Delgado , P. L. ( 2000 ). Depression: The case for a monoamine deficiency . The Journal of Clinical Psychiatry , 61 ( Suppl 6 ), 7 – 11 . https://pubmed.ncbi.nlm.nih.gov/10775018/ 10775018

[b14] Delva , N. C. , & Stanwood , G. D. ( 2021 ). Dysregulation of brain dopamine systems in major depressive disorder . Experimental Biology and Medicine , 246 ( 9 ), 1084 . 10.1177/1535370221991830 33593109 PMC8113739

[b15] de M. Lyra e Silva , N. , Lam , M. P. , Soares , C. N. , Munoz , D. P. , Milev , R. , & De Felice , F. G. ( 2019 ). Insulin resistance as a shared pathogenic mechanism between depression and type 2 diabetes . Frontiers in Psychiatry/Frontiers Research Foundation , 10 , 57 . 10.3389/fpsyt.2019.00057 PMC6382695 30837902

[b16] Devor , A. , Andreassen , O. A. , Wang , Y. , Mäki-Marttunen , T. , Smeland , O. B. , Fan , C.-C. , Schork , A. J. , Holland , D. , Thompson , W. K. , Witoelar , A. , Chen , C.-H. , Desikan , R. S. , McEvoy , L. K. , Djurovic , S. , Greengard , P. , Svenningsson , P. , Einevoll , G. T. , & Dale , A. M. ( 2017 ). Genetic evidence for role of integration of fast and slow neurotransmission in schizophrenia . Molecular Psychiatry , 22 ( 6 ), 792 . 10.1038/mp.2017.33 28348379 PMC5495879

[b17] Dima , D. , Modabbernia , A. , Papachristou , E. , Doucet , G. E. , Agartz , I. , Aghajani , M. , Akudjedu , T. N. , Albajes-Eizagirre , A. , Alnaes , D. , Alpert , K. I. , Andersson , M. , Andreasen , N. C. , Andreassen , O. A. , Asherson , P. , Banaschewski , T. , Bargallo , N. , Baumeister , S. , Baur-Streubel , R. , Bertolino , A. , … Frangou , S. ( 2022 ). Subcortical volumes across the lifespan: Data from 18,605 healthy individuals aged 3-90 years . Human Brain Mapping , 43 ( 1 ), 452 – 469 . 10.1002/hbm.25320 33570244 PMC8675429

[b18] Duman , R. S. , Sanacora , G. , & Krystal , J. H. ( 2019 ). Altered connectivity in depression: GABA and glutamate neurotransmitter deficits and reversal by novel treatments . Neuron , 102 ( 1 ), 75 . 10.1016/j.neuron.2019.03.013 30946828 PMC6450409

[b19] Elliott , L. T. , Sharp , K. , Alfaro-Almagro , F. , Shi , S. , Miller , K. L. , Douaud , G. , Marchini , J. , & Smith , S. M. ( 2018 ). Genome-wide association studies of brain imaging phenotypes in UK Biobank . Nature , 562 ( 7726 ), 210 – 216 . 10.1038/s41586-018-0571-7 30305740 PMC6786974

[b20] Espinoza Oyarce , D. A. , Shaw , M. E. , Alateeq , K. , & Cherbuin , N. ( 2020 ). Volumetric brain differences in clinical depression in association with anxiety: A systematic review with meta-analysis . Journal of Psychiatry & Neuroscience: JPN , 45 ( 6 ), 406 . 10.1503/jpn.190156 32726102 PMC7595741

[b21] Feldstein Ewing , S. W. , Bjork , J. M. , & Luciana , M. ( 2018 ). Implications of the ABCD study for developmental neuroscience . Developmental Cognitive Neuroscience , 32 , 161 . 10.1016/j.dcn.2018.05.003 29773510 PMC6436802

[b22] Fiory , F. , Perruolo , G. , Cimmino , I. , Cabaro , S. , Pignalosa , F. C. , Miele , C. , Beguinot , F. , Formisano , P. , & Oriente , F. ( 2019 ). The relevance of insulin action in the dopaminergic system . Frontiers in Neuroscience , 13 , 868 . 10.3389/fnins.2019.00868 31474827 PMC6706784

[b23] Fischl , B. ( 2012 ). FreeSurfer . NeuroImage , 62 ( 2 ), 774 – 781 . 10.1016/j.neuroimage.2012.01.021 22248573 PMC3685476

[b24] Fischl , B. , Salat , D. H. , Busa , E. , Albert , M. , Dieterich , M. , Haselgrove , C. , van der Kouwe , A. , Killiany , R. , Kennedy , D. , Klaveness , S. , Montillo , A. , Makris , N. , Rosen , B. , & Dale , A. M. ( 2002 ). Whole brain segmentation: Automated labeling of neuroanatomical structures in the human brain . Neuron , 33 ( 3 ), 341 – 355 . 10.1016/s0896-6273(02)00569-x 11832223

[b25] Flint , J. , & Kendler , K. S. ( 2014 ). The genetics of major depression . Neuron , 81 ( 3 ), 484 . 10.1016/j.neuron.2014.01.027 24507187 PMC3919201

[b26] Frangou , S. , Shirali , M. , Adams , M. J. , Howard , D. M. , Gibson , J. , Hall , L. S. , Smith , B. H. , Padmanabhan , S. , Murray , A. D. , Porteous , D. J. , Haley , C. S. , Deary , I. J. , Clarke , T.-K. , & McIntosh , A. M. ( 2019 ). Insulin resistance: Genetic associations with depression and cognition in population based cohorts . Experimental Neurology , 316 , 20 . 10.1016/j.expneurol.2019.04.001 30965038 PMC6503941

[b27] Furman , D. J. , Hamilton , J. P. , & Gotlib , I. H. ( 2011 ). Frontostriatal functional connectivity in major depressive disorder . Biology of Mood & Anxiety Disorders , 1 ( 1 ), 11 . 10.1186/2045-5380-1-11 22737995 PMC3384258

[b28] García-Marín , L. M. , Rabinowitz , J. A. , Ceja , Z. , Alcauter , S. , Medina-Rivera , A. , & Rentería , M. E. ( 2022 ). The pharmacogenomics of selective serotonin reuptake inhibitors . Pharmacogenomics , 23 ( 10 ), 597 – 607 . 10.2217/pgs-2022-0037 35673953

[b29] García-Marín , L. M. , Reyes-Pérez , P. , Diaz-Torres , S. , Medina-Rivera , A. , Martin , N. G. , Mitchell , B. L. , & Rentería , M. E. ( 2023 ). Shared molecular genetic factors influence subcortical brain morphometry and Parkinson’s disease risk . NPJ Parkinson’s Disease , 9 ( 1 ), 1 – 10 . 10.1038/s41531-023-00515-y PMC10172359 37164954

[b30] Gilsanz , P. , Karter , A. J. , Beeri , M. S. , Quesenberry , C. P. , Jr, & Whitmer , R. A. ( 2018 ). The bidirectional association between depression and severe hypoglycemic and hyperglycemic events in type 1 diabetes . Diabetes Care , 41 ( 3 ), 446 . 10.2337/dc17-1566 29255060 PMC5829958

[b31] Glahn , D. C. , Curran , J. E. , Winkler , A. M. , Carless , M. A. , Kent , J. W. , Charlesworth , J. C. , Johnson , M. P. , Göring , H. H. H. , Cole , S. A. , Dyer , T. D. , Moses , E. K. , Olvera , R. L. , Kochunov , P. , Duggirala , R. , Fox , P. T. , Almasy , L. , & Blangero , J. ( 2012 ). High dimensional endophenotype ranking in the search for major depression risk genes . Biological Psychiatry , 71 ( 1 ), 6 – 14 . 10.1016/j.biopsych.2011.08.022 21982424 PMC3230692

[b32] Grogans , S. E. , Fox , A. S. , & Shackman , A. J. ( 2022 ). The amygdala and depression: A sober reconsideration . The American Journal of Psychiatry , 179 ( 7 ), 454 . 10.1176/appi.ajp.20220412 35775156 PMC9260949

[b33] Gutiérrez-Rojas , L. , Porras-Segovia , A. , Dunne , H. , Andrade-González , N. , & Cervilla , J. A. ( 2020 ). Prevalence and correlates of major depressive disorder: A systematic review . Brazilian Journal of Psychiatry , 42 ( 6 ), 657 . 10.1590/1516-4446-2020-0650 32756809 PMC7678895

[b34] Hare , B. D. , & Duman , R. S. ( 2020 ). Prefrontal cortex circuits in depression and anxiety: Contribution of discrete neuronal populations and target regions . Molecular Psychiatry , 25 ( 11 ), 2742 . 10.1038/s41380-020-0685-9 32086434 PMC7442605

[b35] Hasler , G. ( 2010 ). Pathophysiology of depression: Do we have any solid evidence of interest to clinicians? World Psychiatry: Official Journal of the World Psychiatric Association , 9 ( 3 ), 155 . 10.1002/j.2051-5545.2010.tb00298.x PMC2950973 20975857

[b36] Hedman , A. M. , van Haren , N. E. M. , Schnack , H. G. , Kahn , R. S. , & Hulshoff Pol , H. E . ( 2011 ). Human brain changes across the life span: A review of 56 longitudinal magnetic resonance imaging studies . Human Brain Mapping , 33 ( 8 ), 1987 – 2002 . 10.1002/hbm.21334 21915942 PMC6870052

[b37] Hibar , D. P. , Stein , J. L. , Renteria , M. E. , Arias-Vasquez , A. , Desrivières , S. , Jahanshad , N. , Toro , R. , Wittfeld , K. , Abramovic , L. , Andersson , M. , Aribisala , B. S. , Armstrong , N. J. , Bernard , M. , Bohlken , M. M. , Boks , M. P. , Bralten , J. , Brown , A. A. , Chakravarty , M. M. , Chen , Q. , … Medland , S. E. ( 2015 ). Common genetic variants influence human subcortical brain structures . Nature , 520 ( 7546 ), 224 – 229 . 10.1038/nature14101 25607358 PMC4393366

[b38] Hong , E. P. , & Park , J. W. ( 2012 ). Sample size and statistical power calculation in genetic association studies . Genomics & Informatics , 10 ( 2 ), 117 . 10.5808/gi.2012.10.2.117 23105939 PMC3480678

[b39] Howard , D. M. , Adams , M. J. , Clarke , T. K. , Hafferty , J. D. , Gibson , J. , Shirali , M. , Coleman , J. R. I. , Hagenaars , S. P. , Ward , J. , Wigmore , E. M. , Alloza , C. , Shen , X. , Barbu , M. C. , Xu , E. Y. , Whalley , H. C. , Marioni , R. E. , Porteous , D. J. , Davies , G. , Deary , I. J. , … McIntosh , A. M. ( 2019 ). Genome-wide meta-analysis of depression identifies 102 independent variants and highlights the importance of the prefrontal brain regions . Nature Neuroscience , 22 ( 3 ), 343 – 352 . 10.1038/s41593-018-0326-7 30718901 PMC6522363

[b40] Howard , D. M. , Adams , M. J. , Shirali , M. , Clarke , T.-K. , Marioni , R. E. , Davies , G. , Coleman , J. R. I. , Alloza , C. , Shen , X. , Barbu , M. C. , Wigmore , E. M. , Gibson , J. , Hagenaars , S. P. , Lewis , C. M. , Ward , J. , Smith , D. J. , Sullivan , P. F. , Haley , C. S. , Breen , G. , … McIntosh , A. M. ( 2018 ). Genome-wide association study of depression phenotypes in UK Biobank identifies variants in excitatory synaptic pathways . Nature Communications , 9 ( 1 ), 1 – 10 . 10.1038/s41467-018-05310-5 PMC6117285 30166530

[b41] Hyde , C. L. , Nagle , M. W. , Tian , C. , Chen , X. , Paciga , S. A. , Wendland , J. R. , Tung , J. Y. , Hinds , D. A. , Perlis , R. H. , & Winslow , A. R. ( 2016 ). Identification of 15 genetic loci associated with risk of major depression in individuals of European descent . Nature Genetics , 48 ( 9 ), 1031 – 1036 . 10.1038/ng.3623 27479909 PMC5706769

[b42] Kang , H. J. , Kawasawa , Y. I. , Cheng , F. , Zhu , Y. , Xu , X. , Li , M. , Sousa , A. M. M. , Pletikos , M. , Meyer , K. A. , Sedmak , G. , Guennel , T. , Shin , Y. , Johnson , M. B. , Krsnik , Ž. , Mayer , S. , Fertuzinhos , S. , Umlauf , S. , Lisgo , S. N. , Vortmeyer , A. , … Šestan , N. ( 2011 ). Spatiotemporal transcriptome of the human brain . Nature , 478 ( 7370 ), 483 . 10.1038/nature10523 22031440 PMC3566780

[b43] Kleinridders , A. , Cai , W. , Cappellucci , L. , Ghazarian , A. , Collins , W. R. , Vienberg , S. G. , Pothos , E. N. , & Ronald Kahn , C. ( 2015 ). Insulin resistance in brain alters dopamine turnover and causes behavioral disorders . Proceedings of the National Academy of Sciences of the United States of America , 112 ( 11 ), 3463 . 10.1073/pnas.1500877112 25733901 PMC4371978

[b44] Kleinridders , A. , & Pothos , E. N. ( 2019 ). Impact of brain insulin signaling on dopamine function, food intake, reward, and emotional behavior . Current Nutrition Reports , 8 ( 2 ), 83 – 91 . 10.1007/s13668-019-0276-z 31001792

[b45] Leday , G. G. R. , Vértes , P. E. , Richardson , S. , Greene , J. R. , Regan , T. , Khan , S. , Henderson , R. , Freeman , T. C. , Pariante , C. M. , Harrison , N. A. , MRC Immunopsychiatry Consortium, Hugh Perry , V. , Drevets , W. C. , Wittenberg , G. M. , & Bullmore , E. T. ( 2018 ). Replicable and coupled changes in innate and adaptive immune gene expression in two case-control studies of blood microarrays in major depressive disorder . Biological Psychiatry , 83 ( 1 ), 70 . 10.1016/j.biopsych.2017.01.021 28688579 PMC5720346

[b46] Li , C. , Liu , S. , Lu , X. , & Tao , F. ( 2019 ). Role of descending dopaminergic pathways in pain modulation . Current Neuropharmacology , 17 ( 12 ), 1176 . 10.2174/1570159x17666190430102531 31182003 PMC7057207

[b47] Li , H.-J. , Qu , N. , Hui , L. , Cai , X. , Zhang , C.-Y. , Zhong , B.-L. , Zhang , S.-F. , Chen , J. , Xia , B. , Wang , L. , Jia , Q.-F. , Li , W. , Chang , H. , Xiao , X. , Li , M. , & Li , Y. ( 2020 ). Further confirmation of netrin 1 receptor (DCC) as a depression risk gene via integrations of multi-omics data . Translational Psychiatry , 10 , 98 . 10.1038/s41398-020-0777-y 32184385 PMC7078234

[b48] Lim , G. Y. , Tam , W. W. , Lu , Y. , Ho , C. S. , Zhang , M. W. , & Ho , R. C. ( 2018 ). Prevalence of depression in the community from 30 countries between 1994 and 2014 . Scientific Reports , 8 , 2861 . 10.1038/s41598-018-21243-x 29434331 PMC5809481

[b49] Loh , P.-R. , Tucker , G. , Bulik-Sullivan , B. K. , Vilhjálmsson , B. J. , Finucane , H. K. , Salem , R. M. , Chasman , D. I. , Ridker , P. M. , Neale , B. M. , Berger , B. , Patterson , N. , & Price , A. L. ( 2015 ). Efficient Bayesian mixed-model analysis increases association power in large cohorts . Nature Genetics , 47 ( 3 ), 284 – 290 . 10.1038/ng.3190 25642633 PMC4342297

[b50] Luscher , B. , & Fuchs , T. ( 2015 ). GABAergic control of depression-related brain states . Advances in Pharmacology , 73 , 97 . 10.1016/bs.apha.2014.11.003 25637439 PMC4784429

[b51] Luscher , B. , Shen , Q. , & Sahir , N. ( 2011 ). The GABAergic deficit hypothesis of major depressive disorder . Molecular Psychiatry , 16 ( 4 ), 383 . 10.1038/mp.2010.120 21079608 PMC3412149

[b52] MacQueen , G. , & Frodl , T. ( 2010 ). The hippocampus in major depression: Evidence for the convergence of the bench and bedside in psychiatric research? Molecular Psychiatry , 16 ( 3 ), 252 – 264 . 10.1038/mp.2010.80 20661246

[b53] Malykhin , N. V. , Carter , R. , Seres , P. , & Coupland , N. J. ( 2010 ). Structural changes in the hippocampus in major depressive disorder: Contributions of disease and treatment . Journal of Psychiatry & Neuroscience: JPN , 35 ( 5 ), 337 . 10.1503/jpn.100002 20731966 PMC2928287

[b54] Manitt , C. , Eng , C. , Pokinko , M. , Ryan , R. T. , Torres-Berrío , A. , Lopez , J. P. , Yogendran , S. V. , Daubaras , M. J. , Grant , A. , Schmidt , E. R. , Tronche , F. , Krimpenfort , P. , Cooper , H. M. , Pasterkamp , R. J. , Kolb , B. , Turecki , G. , Wong , T. P. , Nestler , E. J. , Giros , B. , & Flores , C. ( 2013 ). dcc orchestrates the development of the prefrontal cortex during adolescence and is altered in psychiatric patients . Translational Psychiatry , 3 ( 12 ), e338 . 10.1038/tp.2013.105 24346136 PMC4030324

[b55] Mathias , S. R. , Knowles , E. E. , Kent , J. W. , McKay , D. R. , Curran , J. E. , de Almeida , M. A. , Dyer , T. D. , Göring , H. H. , Olvera , R. L. , Duggirala , R. , Fox , P. T. , Almasy , L. , Blangero , J. , & Glahn , D. C. ( 2016 ). Recurrent major depression and right hippocampal volume: A bivariate linkage and association study . Human Brain Mapping , 37 ( 1 ), 191 – 202 . 10.1002/hbm.23025 26485182 PMC4981180

[b56] Medland , S. E. , Grasby , K. L. , Jahanshad , N. , Painter , J. N. , Colodro-Conde , L. , Bralten , J. , Hibar , D. P. , Lind , P. A. , Pizzagalli , F. , Thomopoulos , S. I. , Stein , J. L. , Franke , B. , Martin , N. G. , & Thompson , P. M. ( 2022 ). Ten years of enhancing neuro-imaging genetics through meta-analysis: An overview from the ENIGMA Genetics Working Group . Human Brain Mapping , 43 ( 1 ), 292 – 299 . 10.1002/hbm.25311 33300665 PMC8675405

[b5] Mesika , A. , Klar , A. , & Falik Zaccai , T. C. ( 2018 ). INSR-related severe syndromic insulin resistance . In GeneReviews ^®^ [Internet] . University of Washington , Seattle . https://www.ncbi.nlm.nih.gov/sites/books/NBK476444/ 29369573

[b57] Mitchell , B. L. , Thorp , J. G. , Evans , D. M. , Nyholt , D. R. , Martin , N. G. , & Lupton , M. K. ( 2020 ). Exploring the genetic relationship between hearing impairment and Alzheimer’s disease . Alzheimer’s & Dementia: The Journal of the Alzheimer's Association , 12 ( 1 ), e12108 . 10.1002/dad2.12108 PMC7517507 33005726

[b58] Moin , A. S. M. , Nandakumar , M. , Diane , A. , Dehbi , M. , & Butler , A. E. ( 2020 ). The role of heat shock proteins in type 1 diabetes . Frontiers in Immunology , 11 , 612584 . 10.3389/fimmu.2020.612584 33584694 PMC7873876

[b59] Morgunova , A. , Pokhvisneva , I. , Nolvi , S. , Entringer , S. , Wadhwa , P. , Gilmore , J. , Styner , M. , Buss , C. , Sassi , R. B. , Hall , G. B. C. , O’Donnell , K. J. , Meaney , M. J. , Silveira , P. P. , & Flores , C. A. ( 2021 ). DCC gene network in the prefrontal cortex is associated with total brain volume in childhood . Journal of Psychiatry & Neuroscience , 46 ( 1 ), E154 . 10.1503/jpn.200081 PMC7955849 33206040

[b60] Mullins , N. , Forstner , A. J. , O’Connell , K. S. , Coombes , B. , Coleman , J. R. I. , Qiao , Z. , Als , T. D. , Bigdeli , T. B. , Børte , S. , Bryois , J. , Charney , A. W. , Drange , O. K. , Gandal , M. J. , Hagenaars , S. P. , Ikeda , M. , Kamitaki , N. , Kim , M. , Krebs , K. , Panagiotaropoulou , G. , … Andreassen , O. A. ( 2021 ). Genome-wide association study of more than 40,000 bipolar disorder cases provides new insights into the underlying biology . Nature Genetics , 53 ( 6 ), 817 – 829 . 10.3410/f.740122937.793585895 34002096 PMC8192451

[b61] Nawaz , M. S. , Einarsson , G. , Bustamante , M. , Gisladottir , R. S. , Walters , G. B. , Jonsdottir , G. A. , Skuladottir , A. T. , Bjornsdottir , G. , Magnusson , S. H. , Asbjornsdottir , B. , Unnsteinsdottir , U. , Sigurdsson , E. , Jonsson , P. V. , Palmadottir , V. K. , Gudjonsson , S. A. , Halldorsson , G. H. , Ferkingstad , E. , Jonsdottir , I. , Thorleifsson , G. , … Stefansson , K. ( 2022 ). Thirty novel sequence variants impacting human intracranial volume . Brain Communications , 4 ( 6 ), fcac271 . 10.1093/braincomms/fcac271 36415660 PMC9677475

[b62] Ohi , K. , Shimada , T. , Kataoka , Y. , Yasuyama , T. , Kawasaki , Y. , Shioiri , T. , & Thompson , P. M. ( 2020 ). Genetic correlations between subcortical brain volumes and psychiatric disorders . The British Journal of Psychiatry: The Journal of Mental Science , 216 ( 5 ), 280 – 283 . 10.1192/bjp.2019.277 32000869

[b63] Ormel , J. , Hartman , C. A. , & Snieder , H. ( 2019 ). The genetics of depression: Successful genome-wide association studies introduce new challenges . Translational Psychiatry , 9 ( 1 ), 1 – 10 . 10.1038/s41398-019-0450-5 30877272 PMC6420566

[b64] Pandya , M. , Altinay , M. , Malone , D. A. , Jr , & Anand , A. ( 2012 ). Where in the brain is depression? Current Psychiatry Reports , 14 ( 6 ), 634 . 10.1007/s11920-012-0322-7 23055003 PMC3619732

[b65] Payankaulam , S. , Raicu , A.-M. , & Arnosti , D. N. ( 2019 ). Transcriptional regulation of INSR, the insulin receptor gene . Genes , 10 ( 12 ), 984 . 10.3390/genes10120984 31795422 PMC6947883

[b66] Pickrell , J. K. , Berisa , T. , Liu , J. Z. , Ségurel , L. , Tung , J. Y. , & Hinds , D. A. ( 2016 ). Detection and interpretation of shared genetic influences on 42 human traits . Nature Genetics , 48 ( 7 ), 709 – 717 . 10.1038/ng.3570 27182965 PMC5207801

[b67] Pizzagalli , D. A. , & Roberts , A. C. ( 2022 ). Prefrontal cortex and depression . Neuropsychopharmacology: Official Publication of the American College of Neuropsychopharmacology , 47 ( 1 ), 225 – 246 . 10.1038/s41386-021-01101-7 34341498 PMC8617037

[b68] Rentería , M. E. , Hansell , N. K. , Strike , L. T. , McMahon , K. L. , de Zubicaray , G. I. , Hickie , I. B. , Thompson , P. M. , Martin , N. G. , Medland , S. E. , & Wright , M. J. ( 2014 ). Genetic architecture of subcortical brain regions: Common and region-specific genetic contributions . Genes, Brain, and Behavior , 13 ( 8 ), 821 – 830 . 10.1111/gbb.12177 25199620 PMC4241157

[b69] Reyes-Pérez , P. , García-Marín , L. M. , Aman , A. M. , Antar , T. , Flores-Ocampo , V. , Mitchell , B. L. , Medina-Rivera , A. , & Rentería , M. E. ( 2024 ). Investigating the shared genetic etiology between Parkinson’s disease and depression . Journal of Parkinson’s Disease , 14 ( 3 ), 483 – 493 . 10.3233/jpd-230176 PMC11091633 38457145

[b70] Roddy , D. W. , Farrell , C. , Doolin , K. , Roman , E. , Tozzi , L. , Frodl , T. , O’Keane, & O’Hanlon , E. ( 2019 ). The hippocampus in depression: More than the sum of its parts? Advanced hippocampal substructure segmentation in depression . Biological Psychiatry , 85 ( 6 ), 487 – 497 . 10.1016/j.biopsych.2018.08.021 30528746

[b71] Russo , S. J. , & Nestler , E. J. ( 2013 ). The brain reward circuitry in mood disorders . Nature Reviews. Neuroscience , 14 ( 9 ), 609 – 625 . 10.1038/nrn3381 23942470 PMC3867253

[b72] Satizabal , C. L. , Adams , H. H. H. , Hibar , D. P. , White , C. C. , Knol , M. J. , Stein , J. L. , Scholz , M. , Sargurupremraj , M. , Jahanshad , N. , Roshchupkin , G. V. , Smith , A. V. , Bis , J. C. , Jian , X. , Luciano , M. , Hofer , E. , Teumer , A. , van der Lee , S. J. , Yang , J. , Yanek , L. R. , … Arfan Ikram , M. ( 2019 ). Genetic architecture of subcortical brain structures in 38,851 individuals . Nature Genetics , 51 ( 11 ), 1624 . 10.1038/s42003-019-0537-9 31636452 PMC7055269

[b73] Schell , M. , Wardelmann , K. , & Kleinridders , A. ( 2021 ). Untangling the effect of insulin action on brain mitochondria and metabolism . Journal of Neuroendocrinology , 33 ( 4 ), e12932 . 10.1111/jne.12932 33506556

[b74] Schmaal , L. , Veltman , D. J. , van Erp , T. G. , Sämann , P. G. , Frodl , T. , Jahanshad , N. , Loehrer , E. , Tiemeier , H. , Hofman , A. , Niessen , W. J. , Vernooij , M. W. , Ikram , M. A. , Wittfeld , K. , Grabe , H. J. , Block , A. , Hegenscheid , K. , Völzke , H. , Hoehn , D. , Czisch , M. , … Hibar , D. P. ( 2016 ). Subcortical brain alterations in major depressive disorder: Findings from the ENIGMA Major Depressive Disorder working group . Molecular Psychiatry , 21 ( 6 ), 806 – 812 . 10.1038/mp.2015.69 26122586 PMC4879183

[b75] Sheline , Y. I. ( 2011 ). Depression and the hippocampus: Cause or effect? Biological Psychiatry , 70 ( 4 ), 308 . 10.1016/j.biopsych.2011.06.006 21791257 PMC3733566

[b76] Smith , D. J. , Nicholl , B. I. , Cullen , B. , Martin , D. , Ul-Haq , Z. , Evans , J. , Gill , J. M. R. , Roberts , B. , Gallacher , J. , Mackay , D. , Hotopf , M. , Deary , I. , Craddock , N. , & Pell , J. P. ( 2013 ). Prevalence and characteristics of probable major depression and bipolar disorder within UK Biobank: Cross-sectional study of 172,751 participants . PLoS One , 8 ( 11 ), e75362 . 10.1371/journal.pone.0075362 24282498 PMC3839907

[b77] Stouffer , M. A. , Woods , C. A. , Patel , J. C. , Lee , C. R. , Witkovsky , P. , Bao , L. , Machold , R. P. , Jones , K. T. , de Vaca , S. C. , Reith , M. E. A. , Carr , K. D. , & Rice , M. E. ( 2015 ). Insulin enhances striatal dopamine release by activating cholinergic interneurons and thereby signals reward . Nature Communications , 6 , 8543 . 10.1038/ncomms9543 PMC4624275 26503322

[b78] Sullivan , P. F. , Agrawal , A. , Bulik , C. M. , Andreassen , O. A. , Børglum , A. D. , Breen , G. , Cichon , S. , Edenberg , H. J. , Faraone , S. V. , Gelernter , J. , Mathews , C. A. , Nievergelt , C. M. , Smoller , J. W. , & O’Donovan , M. C. ( 2018 ). Psychiatric genomics: An update and an agenda . The American Journal of Psychiatry , 175 ( 1 ), 15 – 27 . 10.1176/appi.ajp.2017.17030283 28969442 PMC5756100

[b79] Tafet , G. E. , & Nemeroff , C. B. ( 2016 ). The links between stress and depression: Psychoneuroendocrinological, genetic, and environmental interactions . The Journal of Neuropsychiatry and Clinical Neurosciences , 28 ( 2 ), 77 – 88 . 10.1176/appi.neuropsych.15030053 26548654

[b80] Taliaz , D. , Spinrad , A. , Barzilay , R. , Barnett-Itzhaki , Z. , Averbuch , D. , Teltsh , O. , Schurr , R. , Darki-Morag , S. , & Lerer , B. ( 2021 ). Optimizing prediction of response to antidepressant medications using machine learning and integrated genetic, clinical, and demographic data . Translational Psychiatry , 11 ( 1 ), 1 – 9 . 10.1038/s41398-021-01488-3 34238923 PMC8266902

[b81] Tartt , A. N. , Mariani , M. B. , Hen , R. , John Mann , J. , & Boldrini , M. ( 2022 ). Dysregulation of adult hippocampal neuroplasticity in major depression: Pathogenesis and therapeutic implications . Molecular Psychiatry , 27 ( 6 ), 2689 . 10.1038/s41380-022-01520-y 35354926 PMC9167750

[b82] The GTEx Consortium* . ( 2013 ). The Genotype-Tissue Expression (GTEx) project . Nature Genetics , 45 ( 6 ), 580 . 10.1038/ng.3969 23715323 PMC4010069

[b83] Thompson , P. M. ( 2015 ). Cracking the brain’s genetic code . Proceedings of the National Academy of Sciences of the United States of America , 112 ( 50 ), 15269 – 15270 . 10.1073/pnas.1520702112 26582794 PMC4687571

[b84] Thompson , P. M. , Jahanshad , N. , Ching , C. R. K. , Salminen , L. E. , Thomopoulos , S. I. , Bright , J. , Baune , B. T. , Bertolín , S. , Bralten , J. , Bruin , W. B. , Bülow , R. , Chen , J. , Chye , Y. , Dannlowski , U. , de Kovel , C. G. F. , Donohoe , G. , Eyler , L. T. , Faraone , S. V. , Favre , P. , … Zelman , V. ( 2020 ). ENIGMA and global neuroscience: A decade of large-scale studies of the brain in health and disease across more than 40 countries . Translational Psychiatry , 10 ( 1 ), 1 – 28 . 10.1016/j.biopsych.2020.02.167 32198361 PMC7083923

[b85] Thompson , P. M. , Stein , J. L. , Medland , S. E. , Hibar , D. P. , Vasquez , A. A. , Renteria , M. E. , Toro , R. , Jahanshad , N. , Schumann , G. , Franke , B. , Wright , M. J. , Martin , N. G. , Agartz , I. , Alda , M. , Alhusaini , S. , Almasy , L. , Almeida , J. , Alpert , K. , Andreasen , N. C. , … Drevets , W. ( 2014 ). The ENIGMA Consortium: Large-scale collaborative analyses of neuroimaging and genetic data . Brain Imaging and Behavior , 8 ( 2 ), 153 – 182 . 10.1007/s11682-013-9269-5 24399358 PMC4008818

[b86] Torres-Berrío , A. , Hernandez , G. , Nestler , E. J. , & Flores , C. ( 2020 ). The Netrin-1/DCC guidance cue pathway as a molecular target in depression: Translational evidence . Biological Psychiatry , 88 ( 8 ), 611 . 10.1016/j.biopsych.2020.04.025 32593422 PMC7529861

[b87] Torres-Berrío , A. , Lopez , J. P. , Bagot , R. C. , Nouel , D. , Dal Bo G , Cuesta , S. , Zhu , L. , Manitt , C. , Eng , C. , Cooper , H. M. , Storch , K. F. , Turecki , G. , Nestler , E. J. , & Flores , C. ( 2017 ). DCC confers susceptibility to depression-like behaviors in humans and mice and is regulated by miR-218 . Biological Psychiatry , 81 ( 4 ), 306 – 315 . 10.1016/j.biopsych.2016.08.017 27773352 PMC5239724

[b88] Trubetskoy , V. , Pardiñas , A. F. , Qi , T. , Panagiotaropoulou , G. , Awasthi , S. , Bigdeli , T. B. , Bryois , J. , Chen , C.-Y. , Dennison , C. A. , Hall , L. S. , Lam , M. , Watanabe , K. , Frei , O. , Ge , T. , Harwood , J. C. , Koopmans , F. , Magnusson , S. , Richards , A. L. , Sidorenko , J. , … O’Donovan , M. C. ( 2022 ). Mapping genomic loci implicates genes and synaptic biology in schizophrenia . Nature , 604 ( 7906 ), 502 – 508 . 10.1038/s41586-022-04434-5 35396580 PMC9392466

[b89] Unverdi , M. , & Alsayouri , K. ( 2023 ). Neuroanatomy, cerebellar dysfunction . In StatPearls [Internet] . StatPearls Publishing . https://www.ncbi.nlm.nih.gov/books/NBK545251/ 31424835

[b90] Videbech , P. , & Ravnkilde , B. ( 2015 ). Hippocampal volume and depression: A meta-analysis of MRI studies . The American Journal of Psychiatry , 161 ( 11 ), 1957 – 1966 . 10.1176/appi.ajp.161.11.1957 15514393

[b91] Vosberg , D. E. , Leyton , M. , & Flores , C. ( 2020 ). The Netrin-1/DCC guidance system: Dopamine pathway maturation and psychiatric disorders emerging in adolescence . Molecular Psychiatry , 25 ( 2 ), 297 . 10.1038/s41380-019-0561-7 31659271 PMC6974431

[b92] Watanabe , K. , Taskesen , E. , & van Bochoven , A. ( 2017 ). Functional mapping and annotation of genetic associations with FUMA . Nature Communications , 8 ( 1 ), 1 – 11 . 10.1038/s41467-017-01261-5 PMC5705698 29184056

[b93] Werme , J. , van der Sluis , S. , & de Leeuw , C. A. ( 2022 ). An integrated framework for local genetic correlation analysis . Nature Genetics , 54 ( 3 ), 274 – 282 . 10.1038/s41588-022-01017-y 35288712

[b94] Wigmore , E. M. , Clarke , T. K. , Howard , D. M. , Adams , M. J. , Hall , L. S. , Zeng , Y. , Gibson , J. , Davies , G. , Fernandez-Pujals , A. M. , Thomson , P. A. , Hayward , C. , Smith , B. H. , Hocking , L. J. , Padmanabhan , S. , Deary , I. J. , Porteous , D. J. , Nicodemus , K. K. , & McIntosh , A. M. ( 2017 ). Do regional brain volumes and major depressive disorder share genetic architecture? A study of Generation Scotland (n = 19 762), UK Biobank (n = 24 048) and the English Longitudinal Study of Ageing (n = 5766) . Translational Psychiatry , 7 ( 8 ), e1205 . 10.1038/tp.2017.148 28809859 PMC5611720

[b95] World Health Organization . ( 2017 ). Depression and other common mental disorders: Global health estimates (No. WHO/MSD/MER/2017.2). World Health Organization. https://apps.who.int/iris/handle/10665/254610

[b96] Wray , N. R. , Ripke , S. , Mattheisen , M. , Trzaskowski , M. , Byrne , E. M. , Abdellaoui , A. , Adams , M. J. , Agerbo , E. , Air , T. M. , Andlauer , T. M. F. , Bacanu , S.-A. , Bækvad-Hansen , M. , Beekman , A. F. T. , Bigdeli , T. B. , Binder , E. B. , Blackwood , D. R. H. , Bryois , J. , Buttenschøn , H. N. , Bybjerg-Grauholm , J. , … Sullivan , P. F. ( 2018 ). Genome-wide association analyses identify 44 risk variants and refine the genetic architecture of major depression . Nature Genetics , 50 ( 5 ), 668 – 681 . 10.1016/j.euroneuro.2017.08.044 29700475 PMC5934326

[b97] Wu , F. , Lu , Q. , Kong , Y. , & Zhang , Z. ( 2023 ). A comprehensive overview of the role of visual cortex malfunction in depressive disorders: Opportunities and challenges . Neuroscience Bulletin , 39 ( 9 ), 1426 . 10.1007/s12264-023-01052-7 36995569 PMC10062279

[b98] Zheng , F. , Liu , G. , Dang , T. , Chen , Q. , An , Y. , Wu , M. , Kong , X. , Qiu , Z. , & Wu , B.-L. ( 2021 ). GABA signaling pathway-associated gene PLCL1 rare variants may be associated with autism spectrum disorders . Neuroscience Bulletin , 37 ( 8 ), 1240 . 10.1007/s12264-021-00707-7 34089506 PMC8353034

[b99] Ziegler , G. , Dahnke , R. , Jäncke , L. , Yotter , R. A. , May , A. , & Gaser , C. ( 2012 ). Brain structural trajectories over the adult lifespan . Human Brain Mapping , 33 ( 10 ), 2377 – 2389 . 10.1002/hbm.21374 21898677 PMC6870331

[b100] Zilaee , M. , & Shirali , S. ( 2016 ). Heat shock proteins and diabetes . Canadian Journal of Diabetes , 40 ( 6 ), 594 – 602 . 10.1016/j.jcjd.2016.05.016 27545596

[b101] Zou , X. H. , Sun , L. H. , Yang , W. , Li , B. J. , & Cui , R. J. ( 2020 ). Potential role of insulin on the pathogenesis of depression . Cell Proliferation , 53 ( 5 ), e12806 . 10.1111/cpr.12806 32281722 PMC7260070

